# Glycerolysis of Poly(lactic acid) as a Way to Extend the “Life Cycle” of This Material

**DOI:** 10.3390/polym11121963

**Published:** 2019-11-29

**Authors:** Marcin Borowicz, Joanna Paciorek-Sadowska, Marek Isbrandt, Łukasz Grzybowski, Bogusław Czupryński

**Affiliations:** Department of Chemistry and Technology of Polyurethanes, Institute of Materials Engineering, Kazimierz, Wielki University, J. K. Chodkiewicza Street 30, 85-064 Bydgoszcz, Poland; m.isbrandt@ukw.edu.pl (M.I.); luk.lukasz.grzybowski@gmail.com (Ł.G.); trzemboguczup@wp.pl (B.C.)

**Keywords:** poly(lactic acid), PLA, glycerolysis, waste, polyol

## Abstract

The article concerns the use of glycerolysis reaction as an alternative method of processing post-production and post-consumer waste from poly(lactic acid) (PLA). Management of waste is a very important issue from an environmental protection and economic point of view. Extending the “life cycle” of PLA is extremely important because it allows to make the most of this material. It also limits economic losses resulting from its disposal in the biodegradation process at the same time. This paper presents a method of glycerolysis of poly(lactic acid) waste using various amounts of anhydrous glycerol (mass ratio from 0.3 to 0.5 parts by weight of glycerol per 1.0 part by weight of PLA). This process was also carried out for pure, unmodified PLA Ingeo^®^ (from NatureWorks) to compare the obtained results. The six liquid oligomeric polyhydric alcohols were obtained as a result of the synthesis. Then, they were subjected to physicochemical tests such as determination of color, smell, density, viscosity, and pH. In addition, the obtained raw materials were subjected to analytical tests such as determination of the hydroxyl value, acid value, water content, and elemental composition. The average molecular weights and dispersity were also tested by gel permeation chromatography (GPC). The assumed chemical structure of the obtained compounds was confirmed by spectroscopic methods such as FTIR, ^1^H NMR, ^13^C NMR. Glycerolysis products were also subjected to differential scanning calorimetry (DSC) to determine thermal parameters. The obtained research results have allowed the precise characterization of newly obtained products and determination of their suitability, e.g., for the synthesis of polyurethane (PUR) materials.

## 1. Introduction

One of the main aims of industrial activities supported by scientific units in the 21st century are actions for sustainable development. The depletion of oil resources, their fluctuations, unstable prices of petrochemical raw materials, and strict environmental protection regulations are currently one of the most important industrial problems. Manufacturers and users of these resources are looking for new sources of raw materials to solve these problems. For example, they tend to use renewable natural resources to completely or partially replace petroleum compounds [1,2]. Petroleum is the basic raw material necessary to produce many materials currently used, among other polymeric materials. These materials have become widely used in everyday life due to their special properties, such as high durability, easy processing, low weight, and low cost of production. They are used almost everywhere e.g., in the food, automotive, pharmaceutical, chemical, cosmetics, and textile industries, etc. There is no doubt that they have permanently entered the human life forming part of it [3].

Plastics can be made both from polymers from fossil sources and renewable sources. Biopolymers and biodegradable polymers are an alternative to petroleum-based polymers, which are accumulated in the natural environment after “end of their life”. Biopolymers are materials obtained from natural resources (e.g., biomass). They are not always biodegradable, despite their natural origin. In turn, biodegradable polymers are materials that can be obtained both from natural (renewable) and fossil (non-renewable) sources. They are degradable in a suitable environment unlike other known polymers, such as polyethylene or polystyrene. However, the increasing production of polymeric materials is also a problem due to the increasing amount of their waste, regardless of their origin. They pose a high risk of increasing environmental pollution with long-term effects that are difficult to predict for all ecosystems. Appropriate manufacture, use, and disposal can contribute to reducing the negative effects associated with the production of plastic products. Therefore, landfilling is undoubtedly the worst way to dispose of waste, both from an economic and ecological point of view [4,5]. It should also be remembered that plastic waste decomposes in a very long time. A well-functioning system of recovery and recycling of polymeric waste is extremely important. It should avoid situations when plastics are massively collected in landfills. On the one hand, it is certain that they will stay there for hundreds of years. On the other hand, these plastics will be still produced using an extremely valuable raw material, which is crude oil. Thus, creating a large number of new polymer waste landfills is now a major environmental problem. The developed and implemented recycling technologies are aimed at limiting the exploitation of fossil raw material sources such as crude oil and minimizing the impact of plastic waste on the environment. The choice of technology is very important and depends on many factors that affect, for example, the costs of the process [6,7,8].

Currently, technology development is focused on improving polymer synthesis methods. New methods often allow the production of plastics that are characterized by high durability, good performance properties, and resistance to chemical and physical agents [9]. Some research on obtaining new biodegradable polymeric materials has started in recent years. This was mainly due to the increasing amount of polymer waste in landfills and thus an increasing problem with the management of it. The biodegradable materials should have functional properties not worse than those obtained by conventional methods. They should be also easily removable from the environment, for example as a result of the composting process. Currently, the industry has made progress in the production of this type of material. They are obtained on an industrial scale both from renewable and petrochemical raw materials [10].

Biodegradable plastics may seem like an obvious solution to the problem of plastic waste, but the current waste management system is not ready for the mass supply of „green” materials. There are few industrial composting plants, and users, in most cases, are not aware of the differences between conventional and biodegradable plastics. Throwing biodegradable plastic waste into waste disposal container of plastics is actually a mistake because it can have a negative impact on the processing of traditional polymer materials. In addition, in the case of biodegradable plastics during their biodegradation in the industrial composting conditions, we do not gain the benefits that we could get during material recycling or energy recovery. Biodegradation processes prevent recovery of the polymers and its re-processing, recovery of monomers and oligomers for their re-use in the polymerization process, and the use of the high calorific value of polymers in the energy recovery process.

The group of biodegradable polymers includes, poly(lactic acid) (PLA). It is obtained mainly by polycondensation of lactic acid (α-hydroxypropionic acid) and by ring-opening polymerization of a cyclic lactide (cyclic di-ester derived from lactic acid). The use of PLA was initially limited to the biomedical sector due to the high cost and relatively low molecular weight of this polymer. Now, it is possible to economically produce high molecular weight PLA while maintaining good mechanical, thermal, and processing properties. PLA is used in many industries, i.e., for production of films, paper lamination, extrusion of thermoformed films, injection molding, and production of a welding layer in multilayer systems. Initially, PLA was mainly used in medicine, in controlled drug delivery and release systems, for the production of orthopedic screws, and in tissue engineering. Poly(lactic acid) is also increasingly used in other fields, such as packaging, textiles, and 3D printing technology. Currently, it is possible to obtain biopolymers or biodegradable polymers with properties similar to conventional polymers (such as polypropylene), due to the development of their production methods [11,12,13,14].

Polylactide, like other biodegradable plastics, does not spontaneously biodegrade in the natural environment. The course of its biodegradation depends on many factors, such as humidity, temperature, pH, the amount and type of microorganisms present in the environment. These factors have a different effect on different polymers. The intensity of their impact on the material can change during the whole process. Therefore, the conditions of the process should be also given, when talking about the biodegradation of the polymer. It is worth noting that only products that easily assimilate with the environment, e.g., carbon dioxide and water, are obtained in the process of complete biodegradation of the material. Therefore, the re-processing of biodegradable polymers is beneficial due to the increasing amount of their waste and gives a measurable economic advantage in comparison with the biodegradation process [15,16,17].

The biodegradation process of polymeric materials has only a social and environmental dimension. The social dimension is primarily related to the increase in competitiveness, which ensures the stability and continuous development of the company that launches innovative products and technologies. It also creates favorable conditions for acquiring new customers. The environmental dimension of biodegradation is associated with the fact that it is a process in which the material decomposes under certain conditions by microorganisms to simple substances found in nature. There are no economic benefits from this type of recycling. An additional problem is the fact that sometimes the level of plastic biodegradation cannot be completely controlled at the product use stage. On the other hand, the chemical recycling process (to which glycerolysis process belongs) has, apart from the social and environmental dimension, also an economic dimension that is very important for companies. The chemical treatment of waste allows to obtain full-value products that can be reused. In addition, it is possible to process the waste stream without quality segregation or purifying, after determining the appropriate reaction parameters. Therefore, chemical recycling implements a sustainable closed cycle model and extends the polymer “life cycle” [18].

The current strategy on polymeric materials focuses on an innovative circular economy that eliminates waste and uses plastics more efficiently [19]. The circular economy is an alternative, more sustainable model in comparison with the linear economy. The principle of a linear economy is “produce, use, discard”. In a circular economy, used products are recycled and reused, unlike in the linear economy approach. The global introduction of such a waste management model will help protect our environment, reduce waste, greenhouse gas emissions, and effectively use existing resources [18].

The aim of the research described in this article was to obtain hydroxyl derivatives that can be a component for the production of e.g., polyurethane materials that can be used in civil engineering or in the automotive industry. The development of an innovative material recycling technology based on PLA waste management is a significant challenge to create a low-carbon, resource-efficient, innovative and competitive circular economy.

## 2. Materials and Methods

### 2.1. Materials

**Figure 1:**
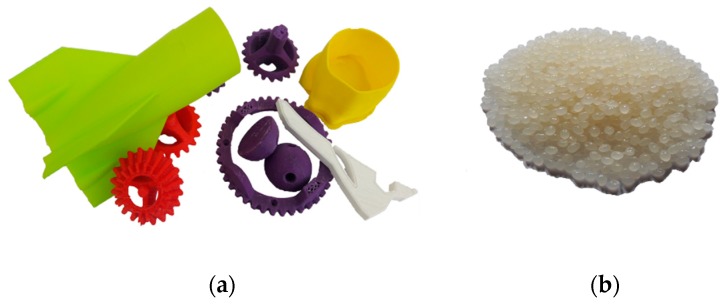
Appearance of: (**a**) poly(lactic acid) waste, (**b**) pure poly(lactic acid).

Poly(lactic acid) waste from 3D printing [wPLA] (Figure 1a) and pure poly(lactic acid) Ingeo^®^ [pPLA] (NatureWorks, Minnetonka, MN, USA) (Figure 1b) were used for the synthesis of new oligomeric polyhydric alcohols in accordance with Polish Patent Application P.424629. New compounds were obtained in the process of transesterification of lactic acid monomers in the PLA macromolecule with anhydrous glycerol (produced by Chempur, Piekary Śląskie, Poland). Glycerolysis reaction catalyst was anhydrous zinc stearate (Chempur, Piekary Śląskie, Poland).

### 2.2. Glycerolysis of Poly(Lactic Acid)

Waste from poly(lactic acid) before the reaction was ground in a laboratory mill to grains with a diameter of less than 5 mm. This operation was necessary so that the polymeric raw material could be placed in the reaction vessel. Pure poly(lactic acid) Ingeo^®^ was in the form of granules with a grain diameter of 5 mm, so there was no need to crush it. Ground waste from poly(lactic acid) or pure poly(lactic acid) was reacted with different amounts of anhydrous glycerol (masses of the reactants are shown in Table 1). The content of the reaction catalyst was always constant and was 0.2 wt.% of polymeric raw material.

**Figure 2:**
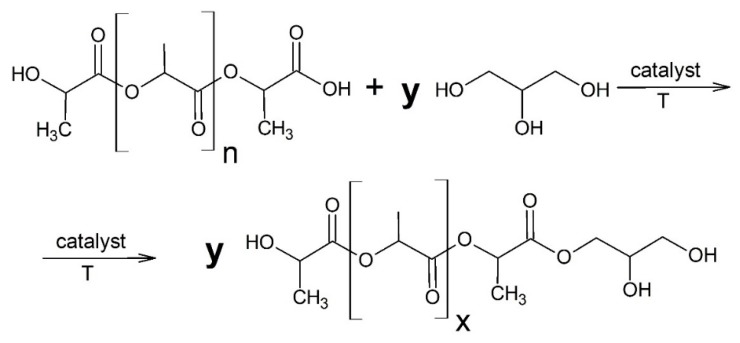
Glycerolysis reaction of poly(lactic acid) [n = y·x].

The synthesis of new oligomeric polyhydric alcohols consisted of putting PLA waste or pure PLA, anhydrous glycerol and zinc stearate into the reactor with a reflux condenser, a thermometer, and a mechanical stirrer. Mass ratios of PLA plastics to glycerol were from 1:0.3 to 1:0.5 (change in glycerol amount by 0.1). The reaction mixture was heated to 200 °C with continuous mixing of the stirrer (700 rpm). Poly(lactic acid) was liquefied in this temperature. The glycerolysis reaction (Figure 2) was carried out for three hours, then, the system was cooled. After cooling, the new compounds were filtered and prepared for testing [20].

### 2.3. Properties of Glycerolysis Products

Physicochemical, analytical and spectroscopic tests were performed on the new oligomeric polyhydric alcohols. The susceptibility of the obtained raw materials to biodegradation in the soil environment was also examined due to the biodegradability of poly(lactic acid). This was for determining its suitability e.g., for the synthesis of polyurethane materials.

#### 2.3.1. Physicochemical Tests

The density of oligomeric polyhydric alcohols was measured at 25 °C (298 K) in an adiabatic pycnometer in accordance with ISO 758:1976 [21].

The viscosity of the PLA-based compounds was determined by using a digital rheometer (Fungilab Inc., New York, NY, USA) at 20 °C (293K). The measurements were carried out by using a standard spindle (DIN-87) working with the bushing (ULA-DIN-87). Maintaining a constant temperature of measurement was provided by a thermostat connected to the water jacket of the sleeve.

The pH value was measured using a microprocessor laboratory pH-meter (ORP/ISO/^O^C) with RS22C connector (Hanna Instruments, Woonsocket, RI, USA).

The color and smell of obtained compounds were tested organoleptically.

#### 2.3.2. Analytical Tests

The hydroxyl value (HV) of PLA-based compounds was determined in accordance with Purinova Ltd. Standards—WT/06/07/PURINOVA, by an acylation method with acetic anhydride in N,N’-dimethylformamide, as a medium. An excess of acetic anhydride after hydrolysis and obtained acetic acid were titrated by using a standard potassium hydroxide solution and phenolphthalein, as an indicator [22].

The acid value (AV) was determined also in accordance with WT/06/07/PURINOVA. The analysis was performed by titration of the sample dissolved in acetone by using the standard solution of potassium hydroxide in ethyl alcohol and phenolphthalein as an indicator [22].

Water content in glycerolysis products was determined by the Carl-Fisher method using a non-pyridine reagent of the trade name Titraqual in accordance with PN-81/C-04959 [23].

Elemental analysis was done by Vario EL III CHNSO analyzer (Elementar, Langenselbold, Germany).

The average molecular weights (M_n_ and M_w_) and the dispersity (D) of the new oligomeric polyhydric alcohols were determined by gel permeation chromatography (GPC) by using a Knauer chromatograph (Knauer GmbH, Berlin, Germany). The apparatus was equipped with thermostated columns and a refractometer detector. The measurements were made on the basis of calibration, by using of polystyrene standards in the range of M_n_ from 162 to 25500 g/mol. The functionality (f) of these compounds was calculated on the basis of HV and average M_w_ of obtained compounds from Equation (1):

(1) $$f=\frac{M_{W}\cdot HV}{56100}$$

#### 2.3.3. Spectroscopy Tests

The PLA-based polyhydric alcohols were tested in Fourier transform infrared (FTIR) spectroscopy by using Nicolet iS10 spectrophotometer (Thermo Fisher Scientific, Waltham, MA, USA) from 400 to 4000 cm^-1^ range and in nuclear magnetic resonance spectroscopy ^1^H NMR and ^13^C NMR by using a NMR Ascend III spectrometer (Brücker, Billerica, MA, USA) with a frequency of 400 MHz, in deuterated chloroform, as a solvent.

#### 2.3.4. Differential Scanning Calorimetry

Analysis of the differential scanning calorimetry (DSC) of PLA-based polyhydric alcohols was carried out using DSC 204 F1 apparatus (Netzsch Analysing & Testing, Selb, Germany) in an atmosphere of inert nitrogen gas. The temperature range of measurement was from −40 °C to 180 °C and the heating rate was 10 °C/min. A sample with a mass of 10 mg was used for the test. The sample was not additionally dried before testing.

#### 2.3.5. Biodegradation Tests

Biodegradation tests of PLA-based polyhydric alcohols were carried out in accordance with ISO 17556:2019 using the OxiTop Control S6 apparatus (WTW-Xylem, Rye Brook, NY, USA), which used a respirometric method to measure the oxygen demand necessary for aerobic biodegradation of polymeric materials in the soil. The measurement of consumed oxygen was presented using the value of biochemical oxygen demand (BOD), which is the number of milligrams of captured oxygen per mass unit of tested polyurethane material [24].

The OxiTop Control S6 apparatus consisted of six glass bottles with a capacity of 510 mL equipped with rubber quivers and measuring heads, which were used to measure BOD. They allowed to measure the pressure in the range of 500 to 1350 hPa with an accuracy of 1% at a temperature of 5 °C to 50 °C. The apparatus also included the OC 110 controller (WTW-Xylem, Rye Brook, NY, USA). It was used for communication between the measuring heads, the user, and the Achat OC computer software (WTW-Xylem, Rye Brook, NY, USA), which was used to interpret the obtained measurement results.

Sifted and dried garden soil with a high humus content and physicochemical parameters such as humidity of 5% (according to ISO 11274 [25]), pH of 6 (according to ISO 10390 [26]), grain diameter below 2 mm (collected in Szczepanowo, Kuyavian-Pomeranian Voivodeship, Poland) was used as a biodegradation environment. The measurement was carried out in a system consisting of 200 mg of tested glycerolysis product, 200 g of soil and 100 g of distilled water. The hermetic-closed system was placed in a laboratory incubator at 20 ± 0.2 °C and thermostated at this temperature for 28 days.

The biochemical oxygen demand (BOD) for a single OxiTop Control S6 bottle was determined from Equation (2) taking into account the BOD of the tested system reduced by the BOD of the soil and concentration of the tested compound in the soil [24,27].

(2) $$BOD_{S}=\frac{BOD_{x}\;-\;BOD_{g}}{c}$$

where: S—number of measurement days, BOD_S_—biochemical oxygen demand of the analyzed sample within S days (mg/L), BOD_x_—biochemical oxygen demand of the measuring system (bottle with sample and soil) (mg/L), BOD_g_—biochemical oxygen demand of soil without a sample (mg/L), c—sample concentration in the tested system (mg/L).

The degree of biodegradation of the polymeric material was determined based on Equation (3):

(3) $$D_{t}=\frac{BOD_{S}}{TOD}\;\cdot 100\%$$

where: D_t_—degree of oligomeric polyhydric alcohol biodegradation (%), TOD – theoretical oxygen demand (mg/L). The theoretical oxygen demand for each system was calculated from Equation (4):

(4) $$TOD=\frac{16\left[2c\;+\;0,5h\;-\;o\right]}{M_{n}}$$

where: c, h, o—mass shares of elements in the molecule of biodegradable material (-), M_n_—molecular weight of biodegradable material (g/mol).

## 3. Results and discussion

### 3.1. Physicochemical Properties

Six new oligomeric polyhydric alcohols were obtained as a result of the glycerolysis reaction. All the obtained compounds were liquids in contrast to the base raw material which was poly(lactic acid). The basic physicochemical properties of the synthesized oligomerols are presented in Table 2.

The color of the obtained compounds was dependent on the color of the poly(lactic acid) used. In the case of products synthesized on the basis of pure PLA, each time a light yellow liquid was obtained. On the other hand, the color of oligomerols based on PLA waste was strongly dependent on the color of waste. Due to the fact that the waste was a mixture of PLA with different colors, the obtained products had different colors like gray, light green, and light brown. The color of these products was the result obtained after mixing all pigments contained in them. All glycerolysis products (both based on waste PLA and pure PLA) did not have any smell. This is due to the absence of any small-molecule products that could cause it. The densities of the oligomeric polyhydric alcohols were similar and ranged from 1.24 to 1.28 g/cm^3^. The obtained values were also close to the densities of the raw materials used for the synthesis (PLA density—1.25 g/cm^3^, anhydrous glycerol density—1.27 g/cm^3^) [28,29]. However, significant differences were observed in the case of the viscosity of the obtained products. The viscosity of final products increased when the glycerol content in the reactant mixture decreased. This was the case for both wPLA and pPLA oligomerols. At a mass ratio of one part by weight of PLA to 0.5 parts by weight of glycerol, the viscosity of the reaction products was 18,870 mPa·s (wPLA500) and 16,420 mPa·s (pPLA500), respectively. The reduction of glycerol in the reaction mixture to 0.3 parts by weight resulted in a 7.5-fold increase in viscosity to 138090 mPa·s for wPLA300 and 124,920 mPa·s for pPLA300, respectively. This increase was mainly due to the change in the length of the obtained oligomerol chains. Short chain products were obtained for a mass ratio of 1:0.5. The decrease in the glycerol content resulted in the preparation of products with longer chains, which directly resulted in an increase in viscosity. Such a high viscosity increase is undesirable from an economic point of view. In industrial practice, high-viscosity raw materials require the use of specialized pumps or additional heating, which allows to decrease this parameter for optimal processing [30,31]. Measurement of pH showed that the value of this parameter for all compounds was in the range of 6.5–6.6. This means that the obtained products were slightly acidic. This was due to the presence of a small amount of carboxyl groups that did not react with glycerol.

### 3.2. Results of Analytical Tests

**Figure 3:**
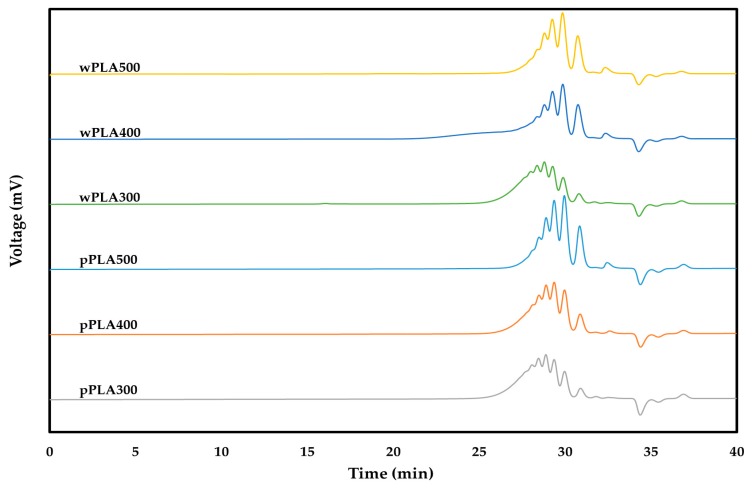
Gel permeation chromatography (GPC) chromatograms of oligomeric polyhydric alcohols based on PLA.

A number of analytical tests were carried out in order to better understand the properties of newly obtained chemical compounds, e.g., determination of hydroxyl value, acid value, water content, elemental composition or molecular weight. The obtained results are presented in Table 3, Table 4 and Table 5 and Figure 3.

The hydroxyl value (HV) is a parameter that indirectly determines the number of hydroxyl groups in the molecule of the analyzed compound. The higher the HV is, the more free hydroxyl groups are in the compound [32]. This parameter has great importance when using polyhydric alcohols (as a polyols) for the production of polyurethane materials. It affects the type of obtained material, e.g., rigid, flexible foam, elastomer, etc., as well as the properties of the obtained material, e.g., mechanical strength [33]. It was noted in the case of glycerolysis products that the higher glycerol content in the reaction mixture caused the higher hydroxyl number of the final product. Products obtained on the basis of a PLA:glycerol ratio of 1:0.5 had an HV of 543.93 mg KOH/g (wPLA500) and 563.27 mg KOH/g (pLA500). However, a 40% decrease in glycerol content also caused a 40% decrease in this parameter to 349.36 mg KOH/g for wPLA300 and 372.87 mg KOH/g for pPLA300, respectively. It is worth noting that the difference between the hydroxyl values of compounds obtained on the basis of poly(lactic acid) waste and compounds based on pure PLA was about 5%. Such small difference meant that the additives and modifiers contained in the PLA waste had practically no effect on the properties of the obtained polyhydric alcohols. This may suggest some kind of repeatability of the obtained products. The acid value of all obtained polyols was about 2 mg KOH/g. This parameter indicates the amount of free carboxyl groups which were in the analyzed compound. The presence of free COOH groups is natural in this case, because each poly(lactic acid) macromolecule ended by this group (Figure 2). There was not esterification reaction of free carboxyl groups with glycerol, because the catalyst (zinc stearate) promoted the transesterification reaction. The water content of all synthesized polyhydric alcohols was in a range from 0.1 to 0.2% wt. This was a relatively small amount that did not require removal by the use of an additional operation, e.g., vacuum distillation. This water was not added into the system in the reaction process because all used reactants were anhydrous. However, it could have been absorbed from the air during cooling or in the filtration process, because both operations were performed in open vessels.

The obtained polyols were also subjected to elemental composition analysis. This study is particularly important when analyzing the mass share of individual elements in a compound. It allows to calculate, e.g., the theoretical oxygen demand (TOD). This parameter is important when calculating the biodegradation degree of the tested material (Section 3.5). The results of the elemental analysis of the obtained compounds based on PLA are presented in Table 4.

It was found, based on the results of the elemental analysis, that the change in the amount of glycerol used for the reaction did not have a significant impact on the changes in the share of carbon, hydrogen, and oxygen in the oligomerols molecules. This was mainly due to the similar elemental composition of lactic acid monomer (C: 40.0%, H: 6.7%, O: 53.3%) and glycerol (C: 39.0%, H: 8.7%, O: 52.3%) [29,34].

The polyhydric alcohols obtained as a result of the reaction of PLA with glycerol were subjected to gel permeation chromatography (GPC). This test was aimed at determining the number average molecular weight (M_n_), the weight average molecular weight (M_w_), and the dispersity (D). The GPC chromatograms are shown in Figure 3. The results obtained based on their analysis are presented in Table 5. In addition, the functionalities of the obtained compounds were calculated based on the hydroxyl values and average molecular weights (Equation (1)).

GPC analysis of the tested samples showed that the number average molecular weight and the weight average molecular weight increased when the glycerol content in the reaction mixture decreased. This is a natural consequence of obtaining longer oligomeric chains by a lower proportion of glycerol. An important parameter of the obtained compounds is their dispersity. This is the ratio of the weight average molecular weight to the number average molecular weight. The dispersity of the obtained polyhydric alcohols was in the range of 1.11–1.17 in all the analyzed cases. This is very advantageous because it is assumed that glycerolysis products can be used as a reactive component for the production of polyurethane materials [35]. High dispersity (>1.8) could significantly reduce or exclude the use of these raw materials in the production of polyurethanes, which should have an ordered structure and have beneficial properties [36,37]. The functionalities of the obtained oligomerols for compounds based on PLA waste and pure PLA were in the range of 2.82–3.20. These results coincide with the assumed functionality for this type of compounds, which should be three due to the presence of one free hydroxyl group derived from the last structural unit of lactic acid and two free hydroxyl groups derived from the glycerol molecule (Figure 2). Oligomeric polyhydric alcohols with a molecular weight below 1000 g/mol and high functionality are used in the production of rigid polyurethane foams and high-performance coatings because they promote the formation of highly crosslinked stiff structures [38,39].

### 3.3. Spectroscopic Analysis

The obtained spectra were interpreted based on a spectroscopic database of pure compounds—Spectral Database for Organic Compounds (SDBS) [40].

**Figure 4:**
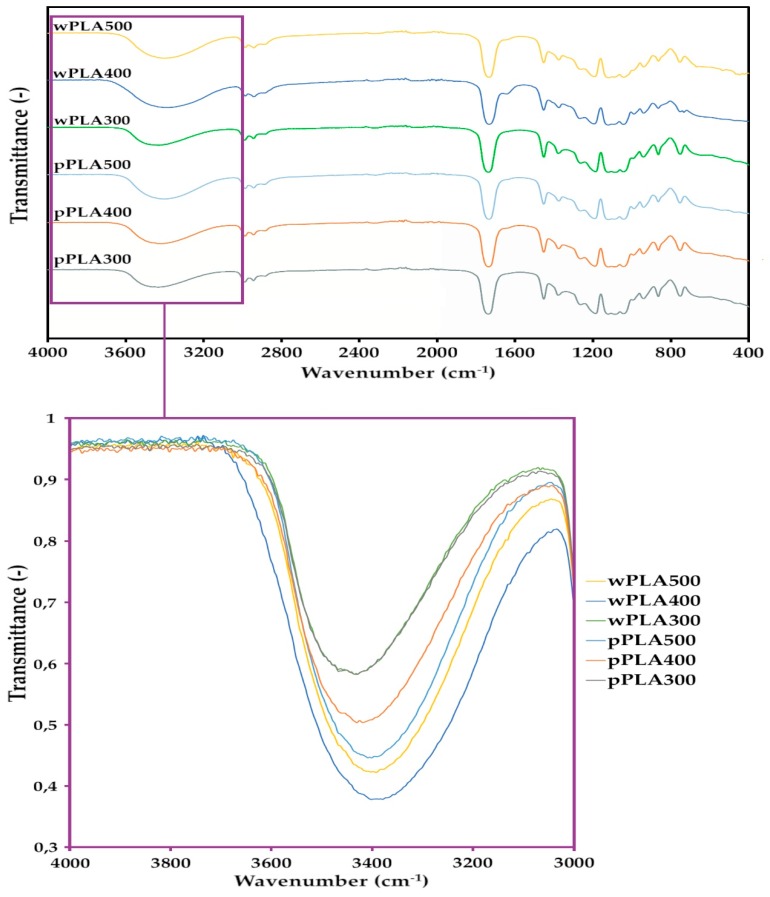
IR spectra of oligomeric polyhydric alcohols based on PLA.

Fourier transform infrared spectroscopy (FTIR) was aimed at confirming the presence of characteristic groups, which were in newly obtained oligomeric polyhydric alcohols. The FTIR spectra for all glycerolysis products are shown in Figure 4.

Analysis of FTIR spectra of PLA-based polyhydric alcohols (Figure 4) showed that there were characteristic bonds of the structure of lactide esters. The spectra of these new compounds showed high band intensity at 3400 cm^−1^, which indicated the presence of O–H bonds in the hydroxyl groups. Bands at 2880–3000 cm^−1^ (stretching) and 1360–1460 cm^−1^ (deformational) belonged to the C–H bond of the –CH_2_– and –CH_3_ group in lactic acid monomers and in glycerol. Bands at 1640–1760 cm^−1^ (stretching) belonged to the C=O bond of the ester group between lactic acid monomers or lactic acid and glycerol, bands at 1050–1270 cm^−1^ (stretching) belonged to the C–O bond of the ester group, and bands at 870–930 cm^−1^ to the free carboxyl group (at the end of the chain of oligomer) that has not reacted with glycerol [41,42,43,44]. Practically, the only changes in the presented FTIR spectra were observed in the wavenumber range of 3200–3400 cm^−1^, i.e., in the characteristic range for the vibrations of O–H bonds of hydroxyl groups. These bands had clearly different intensities. The bands for the wPLA500 and pPLA500 spectra had the highest intensities, while the lowest intensities were for wPLA300 and pPLA300 spectra. This was mainly due to the change in the content of free hydroxyl groups in these compounds. The highest content of these groups was in the compounds in which the highest amount of glycerol was used for the synthesis (PLA500 series). This is consistent with the determination of the hydroxyl values of the obtained oligomeric polyhydric alcohols (Table 3).

**Figure 5:**
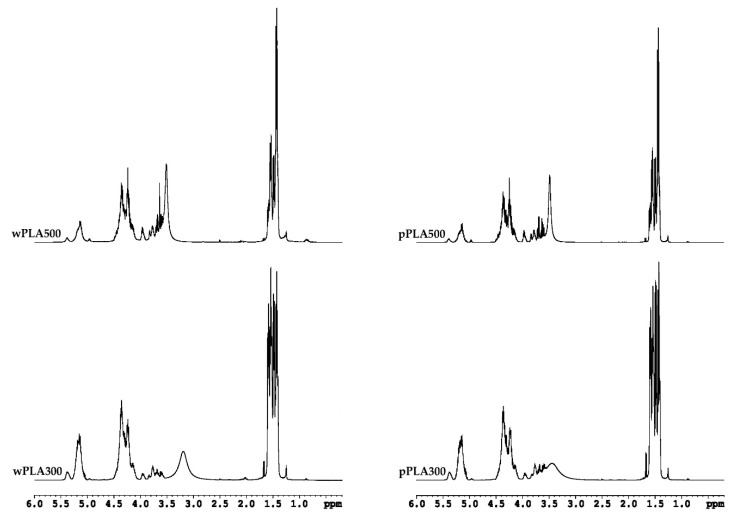
^1^H NMR spectra of oligomeric polyhydric alcohols based on PLA.

**Figure 6:**
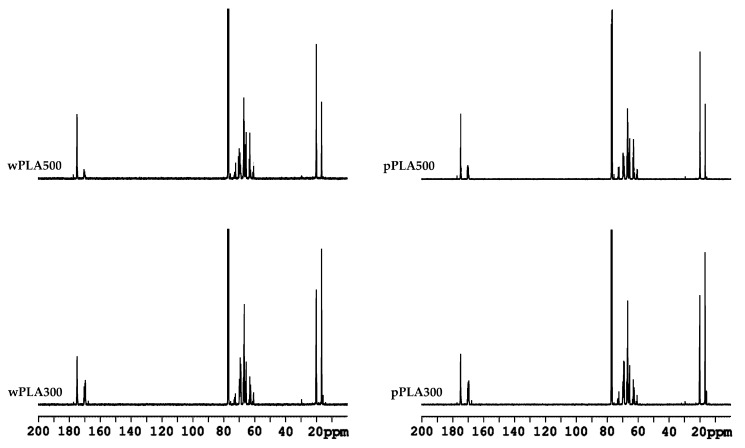
^13^C NMR spectra of oligomeric polyhydric alcohols based on PLA.

Analysis of new PLA-based compounds in nuclear magnetic resonance spectroscopy ^1^H NMR and ^13^C NMR confirmed the expected chemical structure (Figure 2). Selected NMR spectra are presented in Figure 5 and Figure 6.

^1^H NMR spectrum analysis of polyhydric alcohols showed characteristic chemical shifts for 1.40–1.60 ppm protons of methyl group from lactic acid monomers [–CH(C**H**_3_)–], 3.48–3.61 ppm protons of hydroxyl group from lactic acid monomers [**H**O–CH(CH_3_)–], 3.65–3.70 ppm methine protons of glyceryl [–CH_2_–C**H**–CH_2_–], 3.8–3.95 ppm methylene protons of glyceryl [–C**H**_2_–CH–C**H**_2_–], 4.04–4.14 ppm protons of α-CH groups to hydroxyl groups in lactic acid monomers [HO–C**H**(CH_3_)–], 4.32–4.42 ppm protons of hydroxyl groups from glycerol molecules [–O–CH_2_–CH(O**H**)–CH_2_(O**H**)], and 5.13–5.16 ppm protons of α-CH groups to the ester group [–C(O)–O–C**H**(CH_3_)–] [44,45,46,47]. It is worth noting that polyols obtained on the basis of PLA waste and pure PLA have almost identical ^1^H NMR spectra.

^13^C NMR spectrum analysis of oligomers showed characteristic chemical shifts for: 16.61–20.34 ppm carbons of methyl groups [**C**H_3_–], 60.58–63.40 ppm methylene carbons of glyceryl [–**C**H_2_–CH–**C**H_2_–], 65.55–67.45 ppm carbons of α methine group at the hydroxyl group at the end of the lactic acid oligomer chain [>**C**H–OH], 68.98–70.09 ppm carbons of α methine group at the ester group [–C(O)–O–**C**H(CH_3_)–], 72.23–73.30 ppm methine carbons of glyceryl [–CH_2_–**C**H–CH_2_–], 75.66–77.44 ppm carbons from solvent: Deuterated chloroform [**C**DCl_3_], 167.72–170.62 ppm carbons of the carbonyl groups in ester groups [–**C**(O)–O–], and 174.56–177.27 ppm carbons of the carbonyl groups in free carboxyl groups [–**C**(O)–OH] [44,48,49,50]. The ^13^C NMR spectra of products based on PLA waste and pure PLA were practically the same, similar to the ^1^H NMR spectra.

The spectroscopic tests confirmed the assumed structure of transesterification products of poly(lactic acid) by glycerol (Figure 2).

### 3.4. DSC Analysis

**Figure 7:**
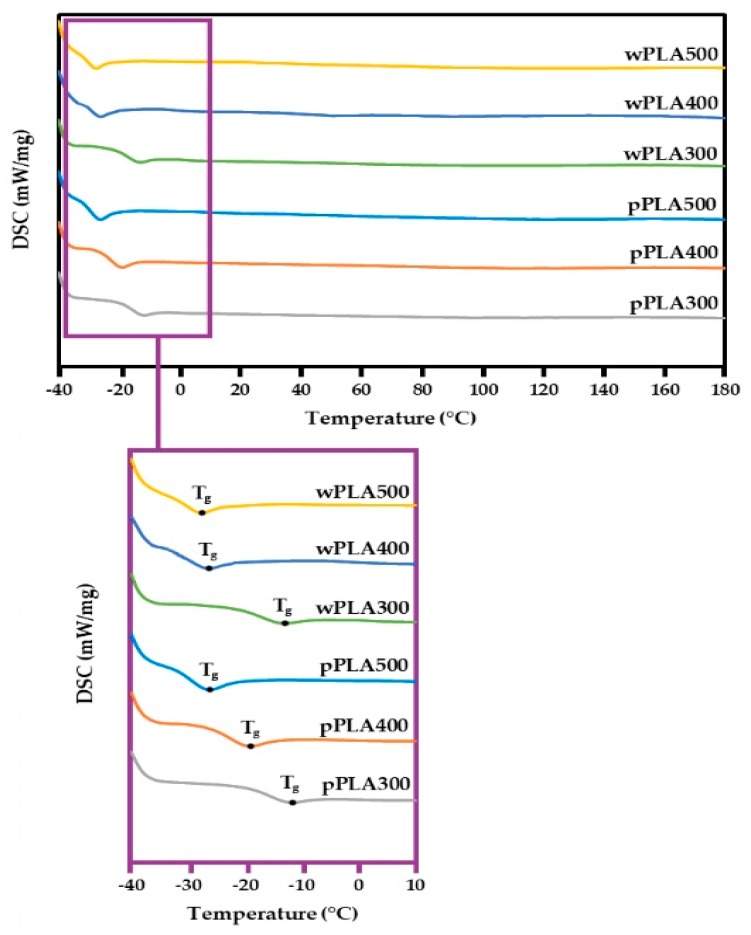
DSC thermograms of oligomeric polyhydric alcohols based on PLA.

Products obtained in the PLA glycerolysis process were also subjected to differential scanning calorimetry (DSC) analysis. This study was intended to determine the possibility of endo- or exothermic changes resulting from elevated temperatures. This measurement is important from the point of application of these raw materials in dosing and mixing devices, e.g., for obtaining polyurethane materials. The absence of chemical changes means the stability of analyzed compounds in the synthesis of PUR materials [51,52]. DSC thermograms are shown in Figure 7.

Differential scanning calorimetry analysis of the obtained compounds in the temperature range from -40 to 180 °C showed the presence of only one transformation peak (P_1_). It was associated with the glass transition of oligmerol. The local extremum of this transformation is the glass transition temperature (T_g_). The transition from glassy state to plastic or liquid state occurs in this temperature. A sudden change in heat capacity and a sudden change in viscosity are observed as a result of this transformation [53,54]. Further heating did not reveal any other endo- or exothermic changes. The thermal properties of polyol raw materials are related to their chemical structure and molecular weight. The presence of additional side chains and functional groups significantly affects the occurrence of individual transformations and the value of their characteristic temperatures. In addition, an increase in the molecular weight of polyhydric alcohols causes an increase in the crystallization degree. Consequently, the mobility of amorphous chains is limited by the neighboring crystal phase. Accordingly, the glass transition temperature may decrease, while the melting point may increase [55]. Table 6 shows the glass transition temperatures of the obtained oligomeric polyhydric alcohols.

The appearance of the glass transition temperature (T_g_) in the negative temperature range was observed in each of the analyzed compounds (Figure 7). The range of glass transition was from −11.8 to −29.2 °C. This temperature changed depending on the glycerol content in the initial reaction mixture. T_g_’s were the lowest at a mass ratio of PLA to glycerol equal to 1:0.5. They were −29.2 °C for wPLA500 and −26.3 °C for pPLA500, respectively. This was mainly due to the presence of shorter and more mobile oligomeric chains that could more easily move relative to each other. The reduction of the glycerol content resulted in increasing glass transition temperatures to −12.3 °C for wPLA300 and −11.8 °C for pPLA300, respectively. This was due to the obtaining of longer and less mobile oligomeric molecules, which could be much more difficult to move relative to each other. The glass transition temperature also depended on the molecular weight. An increase in this parameter (Table 5) resulted in an increase in T_g_. It can be stated based on the differential scanning calorimetry analysis that the obtained compounds are stable at higher temperatures and do not undergo any undesirable changes. This is important when using these glycerolysates for the synthesis of polyurethane materials. Then, they are exposed to high temperatures (e.g., 150 °C when synthesizing rigid polyurethane/polyisocyanurate foams) [56].

### 3.5. Susceptibility to Biodegradation

**Figure 8:**
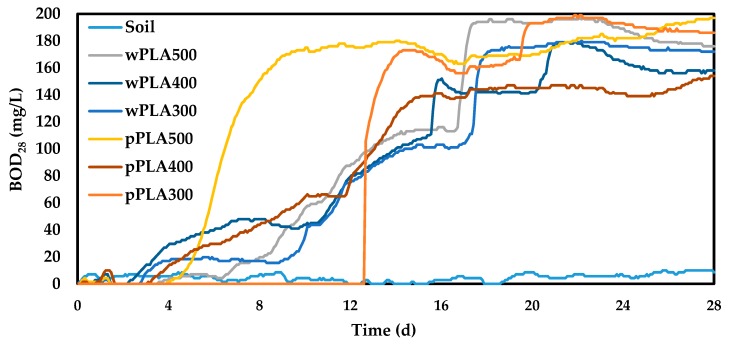
Results of biochemical oxygen demand of PLA-based polyhydric alcohols during 28 days in soil.

A major problem of the current world is the faster and faster increase in the consumption of polymeric materials, and thus also the increase in the production of waste. Poly(lactic acid) belongs to the group of biodegradable polymers of mainly natural origin. Therefore, it is assumed that products obtained on its basis will also be susceptible to biodegradation under forced conditions [57,58]. The biodegradability tests of synthesized polyols in soil environment were conducted using the OxiTop Control S6 apparatus to confirm this assumption. Liquid samples were added to the vessel (bottle of apparatus) in which the soil solution was previously prepared. BOD changes for the analyzed samples were monitored for 28 days. The course of BOD changes during the measurement is shown in Figure 8.

The final BOD values were read for each sample after 28 d. The mass shares of carbon, hydrogen, and oxygen for each polyol were calculated based on elemental analysis results (Table 4). The obtained values are presented in Table 7. The theoretical oxygen demand (TOD) for each PLA-based oligomerol was calculated on their basis. The biodegradation degree (D_t_) of the obtained compounds was calculated using the measured BOD_28_ and the calculated TOD. The results of the calculations are presented in Table 8.

The BOD values were higher than the TOD values for each of the new oligomeric polyhydric alcohols. This meant that the tested compounds could significantly affect the growth of aerobic microorganisms contained in the soil. The standard states that if the BOD value is higher than the TOD value, the tested compounds should be considered as completely biodegradable [24,27,59]. However, it should be remembered that this test is a simplified analysis of biodegradability, because it refers only to environmental parameters and does not take into account the microbiological aspects of the environment used. This is completely sufficient at this stage of research.

## 4. Conclusions

The conditions for poly(lactic acid) (PLA) glycerolysis were developed as part of the research. Glycerolysis of PLA waste and pure PLA was carried out in order to optimize process parameters and properties of final products. New oligomeric polyhydric alcohols were synthesized as a result of the reaction between PLA and anhydrous glycerol using zinc stearate, as a transesterification catalyst. Variable mass ratios of PLA to anhydrous glycerol (in a range from 1:0.3 to 1:0.5) were used during the synthesis. Six new oligomeric polyhydric alcohols were obtained. Next, these compounds were subjected to detailed physicochemical, analytical, and spectroscopic tests. The susceptibility to biodegradation of new oligomerols was also examined in controlled soil environment conditions. The obtained polyhydric alcohols were odorless liquids with comparable densities (about 1.25 g/cm^3^) and pH (about 6.5). Their viscosities were dependent on the mass ratio PLA to glycerol (from 16,000 to 140,000 mPa·s). The reduction of glycerol content in the reaction mixture resulted in a 7.5-fold increase of viscosity value. It was also observed that the glycerol content had an effect on the hydroxyl value. A 40% reduction of glycerol content resulted in a 40% decrease in this parameter from 543.93 mg KOH/g (for wPLA500) and 563.27 mg KOH/g (for pLA500) to 349.36 mg KOH/g (for wPLA300) and 372.87 mg KOH/g (for pPLA300). The difference between hydroxyl values of oligomerols obtained on the basis of waste PLA and pure PLA was insignificant. It was only about 5%. The ratio of reagents and their origin (pure PLA or waste PLA) did not affect the acid value and water content. The acid value was about 2 mg KOH/g, while the water content was in a range from 0.1 to 0.2% wt. for all obtained compounds.

It was found based on the results of GPC analysis of the tested oligomerols, that a decrease in the glycerol content in the reaction mixture caused an increase in the number average molecular weight and the weight average molecular weight. Chemical structure of obtained compounds was confirmed by spectroscopic analysis (FTIR, ^1^H NMR, and ^13^C NMR). Oligomerols obtained on the basis of waste PLA and pure PLA had practically identical ^1^H NMR, ^13^C NMR, and FTIR spectra.

The results of the biodegradation test carried out in a controlled soil environment indicated a very high susceptibility to biodegradation of the obtained glycerolysates. The chemical structure and other physicochemical properties of the obtained polyols indicated that they could be an alternative to petrochemical raw materials used, e.g., in the polyurethane industry. The origin of the raw material, the way it is obtained, and its properties perfectly represent the model of a sustainable circular economy. The obtained compounds are a proposition of an innovative product that can compete with traditional, non-renewable raw materials of petrochemical origin. They also allow more efficient use of existing resources.

## 5. Patents

The synthesis of PLA-based compounds was carried out on the basis of the patented method—Polish Patent Application no. P.424629.

## Figures and Tables

**Table 1:** Amounts of the reactants in glycerolysis process [wPLA—waste from poly(lactic acid), pPLA—pure poly(lactic acid) Ingeo^®^].

Symbol of Compound	wPLA (g)	pPLA (g)	Anhydrous Glycerol (g)	Zinc Stearate (g)
wPLA500	1000.00	0.00	500.00	2.00
wPLA400	1000.00	0.00	400.00	2.00
wPLA300	1000.00	0.00	300.00	2.00
pPLA500	0.00	1000.00	500.00	2.00
pPLA400	0.00	1000.00	400.00	2.00
pPLA300	0.00	1000.00	300.00	2.00

**Table 2:** Physicochemical properties comparison of oligomeric polyhydric alcohols based on poly(lactic acid) (PLA).

Parameter	wPLA500	wPLA400	wPLA300	pPLA500	pPLA400	pPLA300
**Color** **(-)**	grey	light-green	light-brown	light-yellow	light-yellow	light- yellow
**Smell** **(-)**	odorless	odorless	odorless	odorless	odorless	odorless
**Density** **(g/cm^3^)**	1.26 ± 0.03	1.24 ± 0.05	1.26 ± 0.04	1.26 ± 0.04	1.27 ± 0.03	1.28 ± 0.04
**Viscosity (mPa·s)**	18,870 ± 350	39,900 ± 430	138,090 ± 0.04	16,420 ± 210	41,550 ± 320	124,920 ± 760
**pH** **(-)**	6.5 ± 0.1	6.5 ± 0.1	6.6 ± 0.1	6.5 ± 0.1	6.6 ± 0.1	6.6 ± 0.1

**Table 3:** Comparison of basic analytical results of oligomeric polyhydric alcohols based on PLA.

Parameter	wPLA500	wPLA400	wPLA300	pPLA500	pPLA400	pPLA300
**Hydroxyl value (mg KOH/g)**	543.93 ± 6.12	434.36 ± 4.91	349.36 ± 4.11	563.27 ± 5.23	449.96 ± 3.91	372.87 ± 3.19
**Acid value (mg KOH/g)**	1.37 ± 0.10	1.97 ± 0.11	1.93 ± 0.09	2.06 ± 0.07	1.89 ± 0.09	1.87 ± 0.10
**Water content (% wt.)**	0.17 ± 0.01	0.19 ± 0.02	0.20 ± 0.02	0.10 ± 0.01	0.18 ± 0.01	0.20 ± 0.02

**Table 4:** Results of elemental analysis of oligomeric polyhydric alcohols based on PLA.

Element	wPLA500	wPLA400	wPLA300	pPLA500	pPLA400	pPLA300
**C (%)**	39.68 ± 0.19	39.82 ± 0.22	40.07 ± 0.12	39.59 ± 0.17	39.71 ± 0.12	39.98 ± 0.15
**H (%)**	7.43 ± 0.18	7.28 ± 0.17	7.16 ± 0.18	7.32 ± 0.15	7.19 ± 0.21	7.05 ± 0.12
**O (%)**	52.89 ± 0.15	52.90 ± 0.13	52.77 ± 0.20	53.09 ± 0.22	53.10 ± 0.31	52.97 ± 0.16

**Table 5:** Results of GPC analysis and functionality of oligomeric polyhydric alcohols based on PLA.

Parameter	wPLA500	wPLA400	wPLA300	pPLA500	pPLA400	pPLA300
**Mn (g/mol)**	289	342	384	286	332	376
**Mw (g/mol)**	325	393	452	319	381	438
**D (-)**	1.13	1.15	1.17	1.11	1.15	1.17
**f (-)**	3.15	3.11	2.82	3.20	3.06	2.91

**Table 6:** Glass transition temperatures of obtained compounds.

Sample Symbol	wPLA500	wPLA400	wPLA300	pPLA500	pPLA400	pPLA300
**T_g_ (°C** **)**	−29.2	−26.8	−12.3	−26.3	−19.2	−11.8

**Table 7:** Mass shares of individual elements in PLA-based polyhydric alcohols.

Element	wPLA500	wPLA400	wPLA300	pPLA500	pPLA400	pPLA300
**C (-)**	0.3959	0.3971	0.3998	0.3968	0.3982	0.4007
**H (-)**	0.0732	0.0719	0.0705	0.0743	0.0728	0.0716
**O (-)**	0.5309	0.5310	0.5297	0.5289	0.5290	0.5277

**Table 8:** Results of biodegradability of PLA-based polyhydric alcohols.

Sample Symbol	wPLA500	wPLA400	wPLA300	pPLA500	pPLA400	pPLA300
**BOD_28_ (mg/L)**	167.5	149.5	163.5	188.5	145.5	173.5
**TOD (mg/L)**	164.7	139.8	124.5	167.1	144.0	127.1
**D_t_ (%)**	100 *	100 *	100 *	100 *	100 *	100 *

* a result higher than 100% means complete biodegradation of the tested material.
